# Prediction of a novel RNA binding domain in crocodilepox Zimbabwe Gene 157

**DOI:** 10.1186/2042-5783-1-12

**Published:** 2011-11-21

**Authors:** Nicole S Little, Taylor Quon, Chris Upton

**Affiliations:** 1 Biochemistry and Microbiology, University of Victoria, 213 Petch Building, Ring Road, Victoria, B.C., V8W 3P6, Canada

**Keywords:** Crocodilepox, vaccinia, poxvirus, dsRNA-binding protein, HHpred, virus, interferon, Robetta

## Abstract

**Background:**

Although the crocodilepox virus (CRV) is currently unclassified, phylogenetic analyses suggest that its closest known relatives are molluscum contagiosum virus (MCV) and the avipox viruses. The CRV genome is approximately 190 kb and contains a large number of unique genes in addition to the set of conserved Chordopoxvirus genes found in all such viruses. Upon sequencing the viral genome, others noted that this virus was also unusual because of the lack of a series of common immuno-suppressive genes. However, the genome contains multiple genes of unknown function that are likely to function in reducing the anti-viral response of the host.

**Results:**

By using sensitive database searches for similarity, we observed that gene 157 of CRV-strain Zimbabwe (CRV-ZWE) encodes a protein with a domain that is predicted to bind dsRNA. Domain characterization supported this prediction, therefore, we tested the ability of the Robetta protein structure prediction server to model the amino acid sequence of this protein on a well-characterized RNA binding domain. The model generated by Robetta suggests that CRV-ZWE-157 does indeed contain an RNA binding domain; the model could be overlaid on the template protein structure with high confidence.

**Conclusion:**

We hypothesize that CRV-ZWE-157 encodes a novel poxvirus RNA binding protein and suggest that as a non-core gene it may play a role in host-range determination or function to dampen host anti-viral responses. Potential targets for this CRV protein include the host interferon response and miRNA pathways.

## Background

Crocodilepox virus (CRV) is an unclassified member of the Poxviridae family and its complete genome spans 190,054 base pairs [1]. The genome of this species is approximately 61.1% G+C, similar to the genomes of molluscum contagiosum virus (MCV) and the ORF-like viruses. This relatively high G+C% distinguishes these 3 groups of viruses from other poxviruses; MCV and ORF are in separate genera, and it is likely that CRV will also be placed into a separate genus when officially classified. It is not clear what has driven the genomes of these 3 groups of viruses to become relatively GC-rich while other poxviruses have drifted towards a high A+T content. It is important to note that although an overall A+T% is often used to characterize poxvirus genomes, the individual genes in the viruses vary widely in nucleotide composition; for example CRV and vaccinia virus (VACV) genes range in A+T composition from 24-56% and 54-73%, respectively.

The level of conservation between the ortholog sets from viruses in the various poxvirus genera varies greatly (25-50% aa identity), this reflects varying structural and functional constraints on different proteins. This level of sequence conservation does not hinder identification and inclusion of CRV proteins into poxvirus ortholog sets, but it does create problems when the CRV proteins of unknown function are searched against databases using programs such as BLASTp [2] since the additional aa changes generated by the pressure to switch to G/C nucleotides reduces the percentage aa identity even though chemically similar aa may have been substituted.

Poxviruses encode numerous proteins that block the host anti-viral response, including proteins that resist the actions of both type I and type II interferons (IFN). These viruses not only encode soluble receptors that block IFN activity (orthologs of VACV-Cop B19, B8) and intracellular inhibitors of IFN activity (orthologs of VACV-Cop H1, E3, K3, and C7) but also interfere with host IFN signalling [3,4]. VACV-Cop E3 is a dsRNA binding protein that blocks the action of the IFN-inducible dsRNA-dependent protein kinase (PKR). E3 binds both dsRNA and PKR to inhibit its activity as well as inhibiting other host molecules that are part of IFN signalling [3].

CRV is interesting because of its diversity from other poxviruses, but it also creates significant problems for crocodile farming around the world. Therefore, identifying gene function is of interest in trying to understand how this virus controls host anti-viral responses and for potential vaccine design. Such work can also lead to a better understanding of the reptilian immune system. Therefore we have used a variety of bioinformatics tools to try to predict the function of the many CRV proteins for which the function is currently unknown.

## Results and Discussion

### Similarity searches for remote homologs of CPV unknown proteins

65 CPV proteins annotated as unknown function in the Viral Orthologous Clusters database (VOCs) [5] were searched against the National Center for Biotechnology Information (NCBI) non-redundant protein database using position-specific iterated-BLAST (PSI-BLAST). The only significant hits returned by these searches were self-matches to CRV proteins or matches to other poxvirus orthologs. This was somewhat surprising because of the recent explosive growth in genome sequencing, but may reflect the paucity of sequencing of reptilian or related genomes.

Next, these protein sequences were processed using the remote homology detection server HHPred [6,7] through the PDBalert interface [8], which is a more sensitive tool that uses 2° structure information and makes profile-to-profile searches against structural databases. Only 5 of the unknown CRV proteins generated significant (probability ≥80%, e-value ≤ 0.05) hits in the PDBalert [8] scoring system. For 2 proteins (CRV-ZWE-016 and CRV-ZWE-028) the matches were due to amino acid repeats in the protein sequences. These repeats did not match other protein repeats, but they generated areas rich in a few amino acids and these triggered matches to other proteins with regions in those particular amino acids. One match (CRV-ZWE-076), to the family of flap endonucleases (VACV strain Copenhagen is G5R), had been discovered previously by using PDBalert [8] (but had not been included in the genome GenBank file which is used to populate the VOCs database), as had the similarity between CRV-ZWE-078 and the NLPC/P60 superfamily of proteins [9]. The final significant match, and the focus of this paper, from the PDBalert searches was between CRV-ZWE-157 and a series of proteins containing dsRNA binding domains (DRBP).

### Characterization and Comparative Analysis of CRV-157 and dsRNA binding domains

Prediction of protein function from low similarity alignments, within the so-called twilight zone (<20% identity), is difficult and is best approached by gathering multiple lines of evidence that can build to form a solid conclusion. As determined above using PSI-BLAST, it appears that CRV-ZWE-157 is unique in the known poxviruses and is not an ortholog of the poxvirus PKR inhibitor (VACV-Copenhagen-E3L) that also contains a dsRNA-binding domain. The E3L ortholog of ORF virus, which also possesses a very GC-rich genome, is more than 22% identical to any of the poxvirus orthologs so it is unlikely that CRV-ZWE-157 simply fails to classify with this ortholog family.

When CRV-ZWE-157 was used as input with the PDBalert tool, more than 15 protein hits were matched to a dsRNA-binding domain containing protein, each with a probability score of over 95% and with e-values less than 5.0 × 10^-2^. The significant feature of this result is that many of these proteins were distinct with relatively low identity scores showing that critical, well-conserved, amino acids within these dsRNA binding domains were responsible for the significant scores. Following this discovery, we revisited the PSI-BLAST search results and noted that although the E-values for the top two hits were very poor (>0.85), they were both proteins that contained dsRNA-binding domains (data not shown).

The CRV-ZWE-157 primary sequence was also analysed with InterProScan[10], which searches a protein against a variety of databases containing confirmed and predicted protein motifs/domains. Only the Gene3D and Superfamily tools were able detect a dsRNA binding-like domain in the early region of CRV-157. Ribonuclease III [11], ATP-dependent RNA helicase A [12], Interferon-inducible dsRNA-dependent protein kinase activator A [13], and Maternal effect protein staufen [14] all contain comparable dsRNA-binding domains.

**Figure 1:**
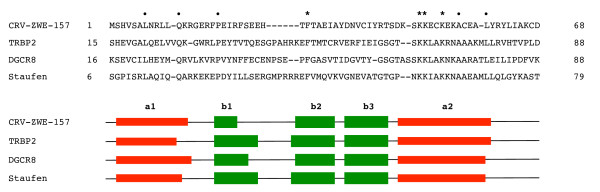
**Multiple alignment of CRV-ZWE-157, RISC-loading complex subunit TRBP2 [PDB:**3ADL**] **[29]**, DGCR8 [PDB:**1X47**] **[45]**, and Staufen RNA binding homolog [PDB:**1UHZ**] **[46]. Upper panel: Only the predicted dsRNA-binding region of CRV-ZWE-157 and the known dsRNA-binding regions of the other proteins are shown. Asterisks show amino acids crucial for dsRNA binding, dots indicate other perfectly conserved residues. Lower panel: Red bars represent α helices and green arrows represent β strands; these are aligned with the amino acid sequences above. The ksdssp algorithm was used to determine the secondary structures from the 3D structures or models.

To appreciate the degree of conservation between the primary sequences of CRV-ZWE-157 and 3 distinct DRBPs from the PDBalert results, they were aligned using multiple sequence comparison by log-expectation (MUSCLE) [15] and manually refined using the Base-By-Base editor [16]. The alignment (Figure 1) revealed that the proteins are only similar over the dsRNA-binding domain (amino acids1-65 of CRV-ZWE-157) and that the percent identity between CRV-ZWE-157 and any of these DRBPs was very low (10-30%). However, the small set of conserved amino acids are in fact conserved among all of the DRBPs including the key residues required for RNA binding. Of the 5 amino acid residues critical to Staufen RNA binding [17], 4 are absolutely conserved in CRV-ZWE-157 and the other proteins (Figure 1). The RNA binding domain of these proteins happens to be at the N-terminus, but frequently they are located at other positions. The RNA binding domain of CRV-ZWE-157 is contained within the first 65 aa of the 127 aa protein. This also differentiates CRV-ZWE-157 from the poxvirus E3L orthologs that have a Z-DNA binding domain at the N-terminus and the dsRNA binding domain at the C-terminus of the protein [18].

To further assess the prediction that CRV-ZWE-157 contains a dsRNA-binding domain, we used BindN+ [19], a tool that predicts nucleic acid binding regions from primary protein sequences. This algorithm also indicated that the N-terminal region of CRV-ZWE-157 could bind RNA and calculated that the sites with the highest RNA binding affinities were located 1) at the end of the third β strand/start of the second α-helix and 2) and the end of the first α-helix. This is in agreement with the later modeling experiment (see below).

The secondary structure of almost all dsRNA-binding domains consists of two α helices that lie on a three-stranded anti-parallel beta sheet to produce a characteristic α-β-β-β-α fold [20]. Jpred [21] predicted this exact pattern in the N-terminus of CRV-ZWE-157 with minor variations in the lengths of the secondary structure units (data not shown).

### Structure Modeling of CRV-157

**Figure 2:**
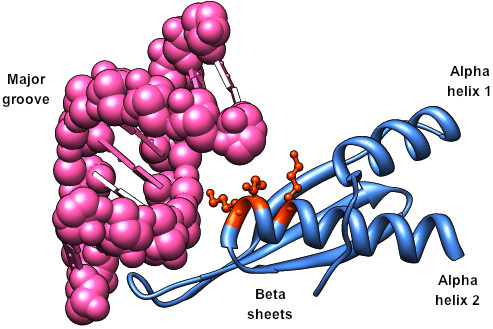
**Predicted structure of CRV-ZWE-157**. A) cartoon representation of Robetta CRV-ZWE-157 model; B-E) electrostatic surface representations of CRV-ZWE-157, TRBP2 [PDB:3ADL] [29], DGCR8 [PDB:2YT4] [25] and dsRNA-specific editase [PDB:2B7V] [47], respectively. Positively and negatively charged surfaces are shown as blue and red, respectively. The electrostatic surface properties were calculated by APBS and the surface diagrams are positioned in the same orientation as (A).

**Figure 3:**
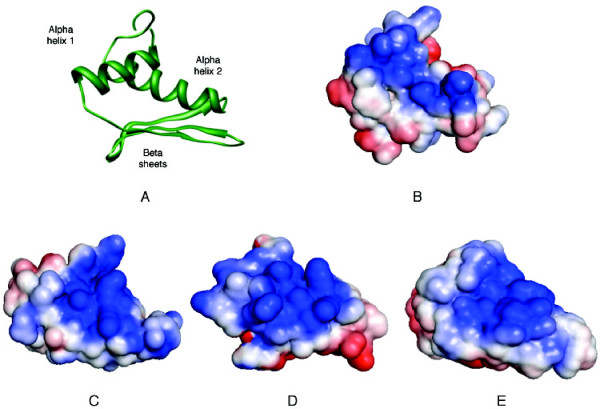
**Structure of RISC-loading complex subunit TRBP2 [PDB:**3ADL**] **[29]**bound to dsRNA**. Protein and RNA are shown as blue and pink, respectively. The three lysine residues in the second alpha helix are indicated in orange with side chains displayed.

Given the preceding support that the CRV-ZWE-157 protein contains a RNA binding domain we submitted the protein sequence to the Robetta structural modeling server [22]. Robetta produced 5 models of CRV-157 (1-127). The 3D models for CRV-ZWE-157 developed from Robetta used the human TAR RNA-binding protein (TRBP; PDB:2CPN) as a reference parent. TRBP is involved in RNA interference (RNAi) and affects the pathway through associations with the enzyme Dicer, which may be involved in the initiation process of RNAi [23]. Dicer requires the association of TRBP, which facilitates the recruitment of siRNA to Ago2 bound by Dicer and miRNA processing by Dicer [24]. DiGeorge syndrome critical region gene 8 (DGCR8), which takes part in the recognition of primary microRNA substrates prior to cleavage ([PDB:2YT4] [25]), was subsequently used for the creation of the full 3D models. However, this does not imply that any particular DRBP is significantly more similar to the CRV protein. The Ginzu module [26] of Robetta that predicts domain architecture of proteins also separated CRV-ZWE-157 into the DRBP-like N-terminal domain and the C-terminal (aa 70-127) domain with a confidence of 2.27, which corresponds to a 96.5% probability in HHSearch [6]; Robetta found no hits for the C-terminal domain. I-TASSER [27,28], another protein structure and function prediction server, supported the choice of DGCR8 ([PDB:2YT4] [25]) as the best model for the N-terminal domain of CRV-ZWE-157. The results of the Robetta prediction are shown in Figure 2 with cartoon and space-filling formats. Both the overall structure and distribution of surface charge on the predicted CRV-ZWE-157 structure are very similar to the known structures of the three DRBPs shown in Figure 3.

The secondary structure of dsRNA-binding domains can be divided into three motifs [29]. The first motif is an α helix, the second motif consists of the three β strands and the last is another α-helix. As shown in Figure 1 (bottom panel) the secondary structure of the CRV-ZWE-157 model agrees very well with the actual secondary structure of known DRBP structures. Several structures of DRBPs bound with RNA have been solved; the first and second domains display a relatively neutral charge and interact with the dsRNA minor groove, while the third domain has a net positive charge that supports interactions with phosphate backbone of the major groove (Figure 3). Substitutions with arginine in this second α helix maintain strong binding to the major groove [30]. Generally, the first α-helix and the β sheet are less well conserved and are thought to act primarily as a scaffold for the second helix by binding to the RNA minor groove [31]. The DRBPs do not bind RNA through specific nucleotide sequences. This is thought to be due to the fact that the interactions with the minor grooves are water-mediated and allow greater flexibility [32].

**Figure 4:**
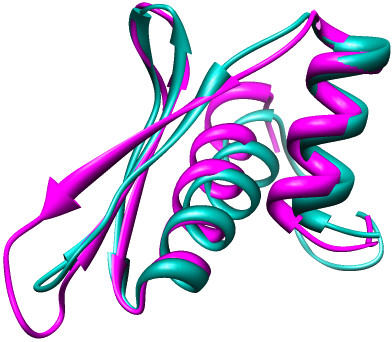
**Superimposition of dsRNA-binding motifs**. The structures of CRV-ZWE-157 (teal) and DGCR8 (purple) were superimposed using the Matchmaker function of Chimera. The RMSD between these two structures was 1.67 Å over 62 alpha carbon pairs.

To provide support of the similarity of the CRV-ZWE-157 model and the known structures we used Chimera [33] to superimpose the CRV and DGCR8 structures (Figure 4). These two structures generated a root mean square deviation (RMSD) of 1.67 Å over 62 alpha carbon pairs whereas the RMSD for CRV and TRBP2 was 1.76 Å over 55 alpha carbon pairs. The RMSD for the superimposition of DGCR8 and TRBP2 DRBPs was only slightly better at 1.502 Å over 63 alpha carbons.

### Genomic localization and Possible Origins of CRV-ZWE-157

As discussed above, the CRV-ZWE-157 protein is not an ortholog of the VACV-Copenhagen E3L RNA-binding protein. The CRV gene is located approximately 25 kb from the right end of the genome in a region mostly populated by hypothetical genes of unknown function that are unique to CRV. Thus, there is little information to be gleaned from the location of this gene to indicate a possible origin. However, it is interesting that the CRV-ZWE-157 gene contains only 51.3% G+C, which is relatively low for the CRV genome, whereas the set of 80 core chordopoxvirus CRV genes have a G+C range of 52-76% with a mean of 64% and the non-core CRV genes have a G+C range of 45-73% and a mean of 59%. Thus, this gene and flanking regions appear to be distinct from the bulk of the genome and the comparatively low G+C content of CRV-157 offers support for its acquisition from the host. There are also numerous such regions scattered throughout the genome that may represent areas that have been acquired via horizontal transfer of DNA, most probably from a host [34].

## Conclusions

This bioinformatics study has gathered several lines of evidence to support the hypothesis that the CRV-ZWE-157 protein contains an N-terminal dsRNA-binding motif. HHpred searches matched a number of DRBPs to the CRV protein and Jpred predicted an α-β-β-β-α fold that is standard in all DRBPs. Perhaps the most conclusive evidence is the fact that Robetta successfully modeled the CRV-ZWE-157 amino acid sequence onto the structure of known DRBPs placing the few highly conserved amino acids in the correct location for interactions with dsRNA. The data supports the hypothesis that the CRV-ZWE-157 is unique in poxviruses and that this gene has been acquired from a CRV host since the virus evolved from the last known common ancestral virus.

DsRNA-binding domains are not sequence specific and are often present in association with a variety of other functioning protein domains including those involved in mRNA localization [17], inhibition of interferon activity [35], Z-DNA binding as part of virus pathogenesis [36], and providing platforms for multi-protein complex assembly through WW domains [37,38]. Unfortunately, analysis of the C-terminal half of CRV-ZWE-157 gave no clue as to the possible function of this domain. However, given that this protein is unique among the poxviruses, it is clearly not one of the "essential poxvirus genes". Two possible scenarios are that it functions in a unique host-range capacity or as a novel virulence factor. For the latter, the dsRNA-binding domain suggests possible functions that include the blocking of interferon function, well known in poxviruses, or a role in the processing of miRNAs.

## Methods

All poxvirus genome, gene and protein sequences were obtained from the VOCs database [5]; other protein sequences were downloaded from the NCBI databases. Protein crystal structures models were retrieved from the Protein Data Bank (PDB) and referenced by its PDB ID in parentheses. Searches for remote homologs were initially conducted with PSI-BLAST [2] with subsequent searches using HHPred [6,7] via the PDBalert interface [8]. InterProScan [10] with its associated domain analysis tools was run at the European Bioinformatics Institute web site. Multiple sequence alignments (MSA) of protein sequences were generated using MUSCLE [15], which has been incorporated into the Base-By-Base MSA editor [16]. Complete poxvirus genomes were visualized and analysed using the Viral Genome Organizer (VGO; [39]) tool at the Viral Bioinformatics Resource Centre (VBRC [40]).

Prediction of the secondary structure of CRV-ZWE-157 and other DRBPs was performed with the Jpred secondary structure prediction server [21] and BindN was used to calculate regions that were likely to bind RNA based on amino acid side chain pK_a_, hydrophobicity, and amino acid molecular mass [19]. The Robetta protein structure prediction server [22] was used to determine if the amino acid sequence of CRV-157 could be modeled onto the structure of known DRBPs with confirmation using I-TASSER [27,28]. Protein structures were displayed using Chimera [33] and JMol [41]. Secondary structure information used in the MSA was extracted from the 3D structures using the Kabsch and Sander algorithm for defining the secondary structure of proteins (ksdssp) module [42] of Chimera. The electrostatic surface representations of the CRV-ZWE-157 protein structure and the DRBP template were produced by the Adaptive Poisson-Boltzmann Solver (APBS) plugin [43] in PyMOL [44]

## List of Abbreviations

aa: amino acid; APBS: Adaptive Poisson-Boltzmann Solver; BLASTp: Basic local alignment search tool protein; CRV: Crocodilepox virus; CRV-ZWE: Crocodilepox virus strain Zimbabwe; DGCR8: DiGeorge syndrome critical region gene 8; DRBP: double-stranded RNA-binding proteins; dsRNA: double-stranded RNA; KSDSSP: Kabsch and Sander algorithm for defining the secondary structure of proteins; MCV: Molluscum contagiosum virus; MSA: Multiple sequence alignments; MUSCLE: multiple sequence comparison by log-expectation; NCBI: National Center for Biotechnology Information; PSI-BLAST: Position specific iterated-BLAST; RMSD: Root mean square deviation; RNAi: RNA interference; TRBP: TAR RNA Binding Protein; VACV: Vaccinia virus; VBRC: Viral bioinformatics resource center; VOCs: Viral Orthologous Clusters

## Competing interests

The authors declare that they have no competing interests.

## Authors' contributions

CU conceived the project. NSL and TQ performed the analyses. All three authors contributed to writing the manuscript and have read and approved the manuscript.

## References

[B1] Afonso CL Tulman ER Delhon G Lu Z Viljoen GJ Wallace DB Kutish GF Rock DL Genome of crocodilepox virus J Virol 2006 80 4978 4991 10.1128/JVI.80.10.4978-4991.2006 16641289 PMC1472061

[B2] Altschul SF Gish W Miller W Myers EW Lipman DJ Basic local alignment search tool J Mol Biol 1990 215 3 403 410 2231712 10.1016/S0022-2836(05)80360-2

[B3] Perdiguero B Esteban M The interferon system and vaccinia virus evasion mechanisms Journal of Interferon & Cytokine research 2009 29 9 581 598 10.1089/jir.2009.0073 19708815

[B4] Haga IR Bowie AG Evasion of innate immunity by vaccinia virus Parasitology 2005 130 S11 S25 10.1017/S0031182005008127 16281988

[B5] Upton C Slack S Hunter AL Ehlers A Roper RL Poxvirus Orthologous Clusters: toward Defining the Minimum Essential Poxvirus Genome J Virol 2003 77 7590 7600 10.1128/JVI.77.13.7590-7600.2003 12805459 PMC164831

[B6] Söding J Protein homology detection by HMM-HMM comparison Bioinformatics 2005 21 951 960 10.1093/bioinformatics/bti125 15531603

[B7] Soding J Biegert A Lupas AN The HHpred interactive server for protein homology detection and structure prediction Nucleic Acids Research 2005 33 W244 W248 10.1093/nar/gki408 15980461 PMC1160169

[B8] Agarwal V Remmert M Biegert A Soding J PDBalert: automatic, recurrent remote homology tracking and protein structure prediction BMC Structural Biology 2008 8 51 10.1186/1472-6807-8-51 19025670 PMC2605448

[B9] Senkevich TG Wyatt LS Weisberg AS Koonin EV Moss B A conserved poxvirus NLPC/P60 superfamily protein contributes to vaccinia virus virulence in mice but not to replication in cell culture Virology 2008 374 506 514 10.1016/j.virol.2008.01.009 18281072 PMC2412903

[B10] Quevillon E Silventoinen V Pillai S Harte N Mulder N Apweiler R Lopez R InterProScan: protein domains identifier Nucleic Acids Research 2005 33 W116 W120 10.1093/nar/gki442 15980438 PMC1160203

[B11] The C. elegans Sequencing Consortium Genome Sequence of the Nematode C. elegans: A Platform for Investigating Biology Science 1998 282 2012 2018 9851916 10.1126/science.282.5396.2012

[B12] Lee CG Eki T Okumura K da Costa Soares V Hurwitz J Molecular analysis of the cDNA and genomic DNA encoding mouse RNA helicase A Genomics 1998 47 365 371 10.1006/geno.1997.5139 9480750

[B13] Patel RC Sen GC PACT, a protein activator of the interferon-induced protein kinase, PKR EMBO J 1998 17 4379 4390 10.1093/emboj/17.15.4379 9687506 PMC1170771

[B14] St Johnston D Beuchle D Nüsslein-Volhard C staufen, a gene required to localize maternal RNAs in the Drosophila egg Cell 1991 66 51 63 10.1016/0092-8674(91)90138-O 1712672

[B15] Edgar RC MUSCLE: multiple sequence alignment with high accuracy and high throughput Nucleic Acids Research 2004 32 1792 1797 10.1093/nar/gkh340 15034147 PMC390337

[B16] Brodie R Smith AJ Roper RL Tcherepanov V Upton C Base-By-Base: Single nucleotide-level analysis of whole viral genome alignments BMC Bioinformatics 5 96 96 15253776 10.1186/1471-2105-5-96 PMC481056

[B17] Ramos A Grünert S Adams J Micklem DR Proctor MR Freund S Bycroft M St Johnston D Varani G RNA recognition by a Staufen double-stranded RNA-binding domain EMBO J 2000 19 997 1009 10.1093/emboj/19.5.997 10698941 PMC305639

[B18] Valentine R Smith GL Inhibition of the RNA polymerase III-mediated dsDNA-sensing pathway of innate immunity by vaccinia virus protein E3 J Gen Virol 2010 91 2221 2229 10.1099/vir.0.021998-0 20519457 PMC3052519

[B19] Wang L Brown SJ BindN: a web-based tool for efficient prediction of DNA and RNA binding sites in amino acid sequences Nucleic Acids Res 2006 34 W243 W248 10.1093/nar/gkl298 16845003 PMC1538853

[B20] Ryter JM Schultz SC Molecular basis of double-stranded RNA-protein interactions: structure of a dsRNA-binding domain complexed with dsRNA EMBO J 1998 17 7505 7513 10.1093/emboj/17.24.7505 9857205 PMC1171094

[B21] Cole C Barber JD Barton GJ The Jpred 3 secondary structure prediction server Nucleic Acids Research 2008 36 W197 W201 10.1093/nar/gkn238 18463136 PMC2447793

[B22] Kim DE Chivian D Baker D Protein structure prediction and analysis using the Robetta server Nucleic Acids Res 2004 32 W526 531 10.1093/nar/gkh468 15215442 PMC441606

[B23] Bernstein E Caudy AA Hammond SM Hannon GJ Role for a bidentate ribonuclease in the initiation step of RNA interference Nature 2001 409 363 366 10.1038/35053110 11201747

[B24] Chendrimada TP Gregory RI Kumaraswamy E Norman J Cooch N Nishikura K Shiekhattar R TRBP recruits the Dicer complex to Ago2 for microRNA processing and gene silencing Nature 2005 436 740 744 10.1038/nature03868 15973356 PMC2944926

[B25] Sohn SY Bae WJ Kim JJ Yeom K-H Kim VN Cho Y Crystal structure of human DGCR8 core Nat Struct Mol Biol 2007 14 847 853 10.1038/nsmb1294 17704815

[B26] Chivian D Kim DE Malmstrom L Baker D Automated prediction of domain boundaries in CASP6 targets using Ginzu and RosettaDOM Proteins 2005 61 193 200 10.1002/prot.20737 16187362

[B27] Roy A Kucukural A Zhang Y I-TASSER: a unified platform for automated protein structure and function prediction Nat Protoc 2010 5 725 738 10.1038/nprot.2010.5 20360767 PMC2849174

[B28] Zhang Y Template-based modelling and free modelling by I-TASSER in CASP7 Proteins 2007 69 108 117 10.1002/prot.21702 17894355

[B29] Yang SW Chen H-Y Yang J Machida S Chua N-H Yuan YA Structure of Arabidopsis HYPONASTIC LEAVES1 and its molecular implications for miRNA processing Structure 2010 18 594 605 10.1016/j.str.2010.02.006 20462493 PMC3119452

[B30] Daviet L Erard M Dorin D Duarte M Vaquero C Gatignol A Analysis of a binding difference between the two dsRNA-binding domains in TRBP reveals the modular function of a KR-helix motif Eur J Biochem 2000 267 2419 2431 10.1046/j.1432-1327.2000.01256.x 10759868

[B31] Stefl R Oberstrass FC Hood JL Jourdan M Zimmermann M Skrisovska L Maris C Peng L Hofr C Emeson RB Allain FH-T The solution structure of the ADAR2 dsRBM-RNA complex reveals a sequence-specific readout of the minor groove Cell 2010 143 225 237 10.1016/j.cell.2010.09.026 20946981 PMC2956598

[B32] Tian B Bevilacqua PC Diegelman-Parente A Mathews MB The double-stranded-RNA-binding motif: interference and much more Nat Rev Mol Cell Biol 2004 5 1013 1023 10.1038/nrm1528 15573138

[B33] Pettersen EF Goddard TD Huang CC Couch GS Greenblatt DM Meng EC Ferrin TE UCSF Chimera--a visualization system for exploratory research and analysis J Comput Chem 2004 25 1605 1612 10.1002/jcc.20084 15264254

[B34] Da Silva M Upton C Host-derived pathogenicity islands in poxviruses Virol J 2005 2 30 30 10.1186/1743-422X-2-30 15823205 PMC1087509

[B35] Nanduri S Carpick BW Yang Y Williams BR Qin J Structure of the double-stranded RNA-binding domain of the protein kinase PKR reveals the molecular basis of its dsRNA-mediated activation EMBO J 1998 17 5458 5465 10.1093/emboj/17.18.5458 9736623 PMC1170871

[B36] Kim Y-G Muralinath M Brandt T Pearcy M Hauns K Lowenhaupt K Jacobs BL Rich A A role for Z-DNA binding in vaccinia virus pathogenesis Proc Natl Acad Sci USA 2003 100 6974 6979 10.1073/pnas.0431131100 12777633 PMC165815

[B37] Ingham RJ Colwill K Howard C Dettwiler S Lim CSH Yu J Hersi K Raaijmakers J Gish G Mbamalu G Taylor L Yeung B Vassilovski G Amin M Chen F Matskova L Winberg G Ernberg I Linding R O'Donnell P Starostine A Keller W Metalnikov P Stark C Pawson T WW Domains Provide a Platform for the Assembly of Multiprotein Networks Mol Cell Biol 2005 25 7092 7106 10.1128/MCB.25.16.7092-7106.2005 16055720 PMC1190255

[B38] Shiohama A Sasaki T Noda S Minoshima S Shimizu N Molecular cloning and expression analysis of a novel gene DGCR8 located in the DiGeorge syndrome chromosomal region Biochemical and Biophysical Research Communications 2003 304 184 190 10.1016/S0006-291X(03)00554-0 12705904

[B39] Upton C Hogg D Perrin D Boone M Harris NL Viral genome organizer: a system for analyzing complete viral genomes Virus Research 2000 70 55 64 10.1016/S0168-1702(00)00210-0 11074125

[B40] Viral Bioinformatics Resource Center http://www.virology.ca

[B41] Hanson RM *Jmol *- a paradigm shift in crystallographic visualization J Appl Crystallogr 2010 43 1250 1260 10.1107/S0021889810030256

[B42] Kabsch W Sander C Dictionary of Protein Secondary Structure: Pattern Recognition of Hydrogen-Bonded and geometrical Features Biopolymers 1983 22 2577 10.1002/bip.360221211 6667333

[B43] Baker NA Sept D Joseph S Holst MJ McCammon JA Electrostatics of nanosystems: Application to microtubules and the ribosome Proceedings of the National Academy of Sciences 2001 98 10037 10041 10.1073/pnas.181342398 PMC56910 11517324

[B44] Schrodinger, LLC The PyMOL Molecular Graphics System

[B45] Schmid G Haverkamp F Rechmann J Schwanitz G Zerres K Hansmann M Kowalewski S [Non-immunologic hydrops fetalis (NIHF)--case report of double partial trisomy 15q and 17q resulting from familial translocation 15/17 and cytogenetic findings in 50 cases with hydrops fetalis] Klin Padiatr 1987 199 309 314 10.1055/s-2008-1026810 3657040

[B46] He F Muto Y Obayashi N Shirouzu M Terada T Kigawa T Inoue M Yabui T Aoki M Seki E Matsuda T Hirota H Yoshida M Koboyashi N Tanaka A Osanai T Matsuo Y Hayashizaki Y Yokoyama S Solution structure of dsRNA binding domain in Staufen homolog 2

[B47] Airenne TT Kidron H Nymalm Y Nylund M West G Mattjus P Salminen TA Structural evidence for adaptive ligand binding of glycolipid transfer protein J Mol Biol 2006 355 224 236 10.1016/j.jmb.2005.10.031 16309699

